# Oxygen therapy enhances the systemic inflammatory response in a human model of experimental inflammation

**DOI:** 10.1016/j.ijcha.2025.101846

**Published:** 2025-11-28

**Authors:** P. Tornvall, P. Svensson, J. Alfredsson, L. Jonasson, L. Nilsson, R. Hofmann, SK. Merid

**Affiliations:** a Department of Clinical Science and Education Södersjukhuset, Karolinska Institutet, Stockholm, Sweden; b Department of Health, Medicine and Caring Sciences, Linköping University. ^b^Department of Cardiology, Region Östergötland, Linköping, Sweden

**Keywords:** Myocardial infarction, Oxygen therapy, Inflammatory proteins, Gene expression, Factor analysis, small non-coding RNA

## Abstract

**Introduction:**

Oxygen therapy does not benefit normoxemic patients with suspected myocardial infarction and may instead enhance the inflammatory response triggered by the tissue necrosis caused by the myocardial infarction. In the present study, we tested the hypothesis that oxygen therapy aggravates systemic inflammation in normoxemic healthy individuals in a human model of experimental inflammation.

**Methods:**

Proteomic and gene expression data from healthy subjects vaccinated against Salmonella Typhii and exposed to oxygen therapy or ambient air were investigated. A multi-omics approach with factor analysis to identify common sources of variation in the systemic inflammatory response associated with oxygen exposure was used.

**Results:**

Oxygen therapy showed a statistically nominal tendency toward aggravation determined by ELISA (IL-6) and proximity extension assay (IL-8). The factor analysis revealed a pro-inflammatory feature that included increases in (CXCL 6, 10 and 11) with decreased small nucleolar RNA.

**Conclusion:**

The results indicate that oxygen therapy enhances experimental systemic inflammation. The mechanism is not clear but future studies should address small nucleolar RNA.

## 1 Introduction

Oxygen therapy does not benefit normoxemic patients with suspected myocardial infarction [1] and may instead enhance the inflammatory response triggered by the tissue necrosis caused by the myocardial infarction. In a previous study we observed a non-significant trend toward increased levels of the pro-inflammatory protein Interleukin-6 (IL-6) induced by oxygen therapy during the acute phase of suspected myocardial infarction [2]. However, the results may have been influenced by confounding factors, such as medication administered in the acute setting. Vaccination against Salmonella Typhii (ST) induces a mild systemic inflammation with similar cardiovascular effects, such as endothelial dysfunction, increased vascular stiffness and activation of coagulation factor VII [3,4], as a myocardial infarction. In the present study, we tested the hypothesis that oxygen therapy aggravates systemic inflammation in normoxemic healthy individuals in the experimental setting of ST-vaccination. Thereafter, we used a multi-omics approach with factor analysis to identify common sources of variation in the systemic inflammatory response related to oxygen exposure.

## 2 Methods

Study subjects were recruited at Linköping University through advertisement. Inclusion criteria were male sex and age 18–40 years old. Exclusion criteria included chronic disease, acute illness or trauma within the last 30 days and any medication within the last 10 days. Additional exclusion criteria were present smoking, difficulty to understand written information in Swedish, participation in an interventional study and known allergy to ST-vaccine components. All study subjects were instructed to refrain from physical exercise for 48 h before examination and have their regular breakfast on the day of the tests. After written informed consent, all 24 study subjects were vaccinated against ST by Typhim Vi® (Sanofi Pasteur UK) prior to randomization. Study subjects were randomized in a 1:1 fashion, to either oxygen treatment at a flow rate of 6 L/min delivered by an open face mask for six hours or ambient air. Blood was drawn from an indwelling catheter in the antecubital vein before, 3 and 6 h after vaccination. IL-6 was analyzed in plasma at all time-points by Magnetic Luminex® Performance Assay Human High Sensitivity Cytokine Base Kit A (RnD systems, Minneapolis, US). A total of 92 immunological protein markers were analyzed in the plasma samples collected before and 6 h after vaccination by proximity extension assay using Olink Proseek® Multiplex Inflammation I Assay (Olink Bioscience AB, Uppsala, Sweden). For gene expression, leucocytes were collected in CPT tubes before and 6 h after vaccination and prepared according to the manufacturer’s instructions prior to lysis (RLTRNA-buffer, Qiagen, Hilden, Germany) and RNA extraction (Allprep DNA/RNA Mini Kit, RNeasy Plus Mini Kit, Qiagen). RNA was analyzed using Affymetrix Human Transcriptome Array 2.0 (Thermo Fisher Scientific, Waltham, MA, US).

The protocol was approved by the local ethics committee (DNR 2017/252-31) and the Swedish medical product agency (EudraCT number 2014-002282-30). All study subjects gave written informed consent.

### 2.1 Statistics

**Fig. 1:**
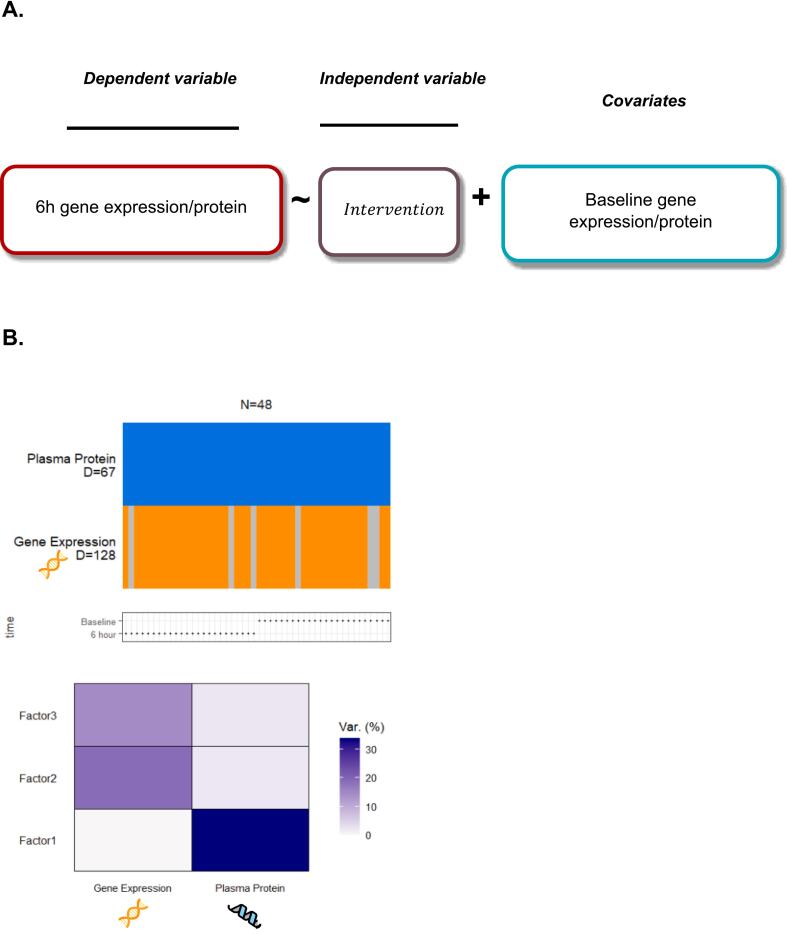
Panel A: Design of regression analysis. Panel B: Design and numbers of analytes included in multi-omics factor analysis.

IL-6 before, 3 and 6 h after vaccination was analyzed by repeated measurement ANOVA. Multi-omics analysis using multi-omics factor analysis version (MOFA2) before and 6 h after vaccination was performed to uncover latent factors that explain variability across gene and protein expression datasets. MOFA 2 was trained using its default parameters. Differential expression analyses for both transcriptomic and proteomic data were conducted using linear regression models implemented in the limma R package. The same statistical framework was applied to evaluate associations between the identified MOFA2 latent factors [5,6] (Fig. 1A).

## 3 Results

**Fig. 2:**
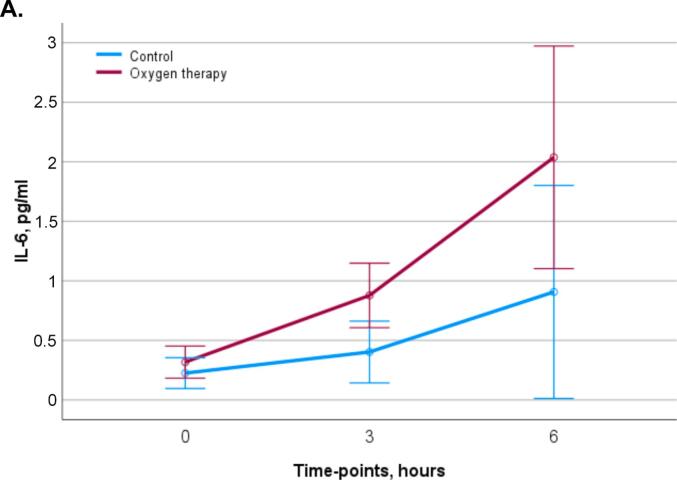

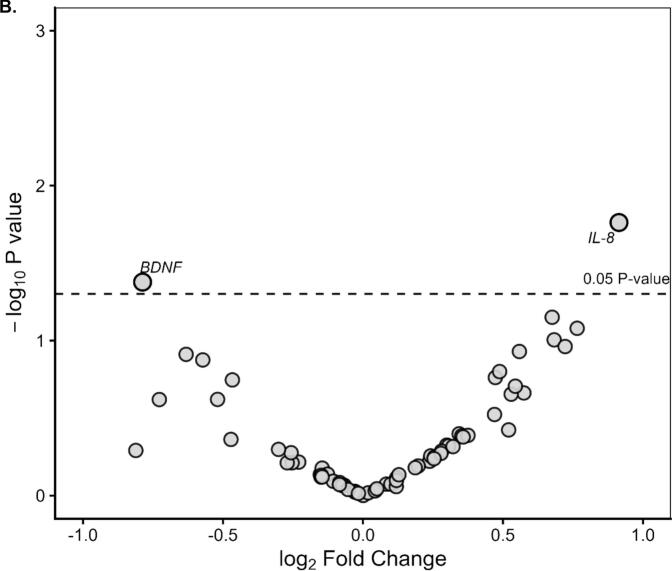

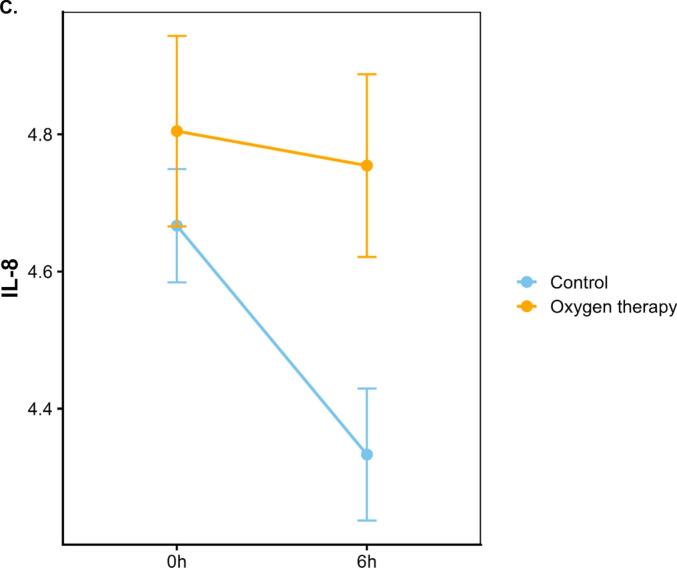

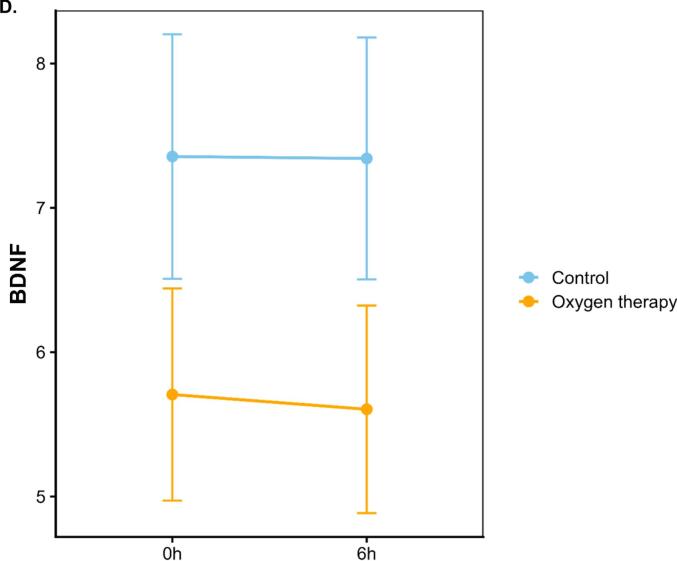

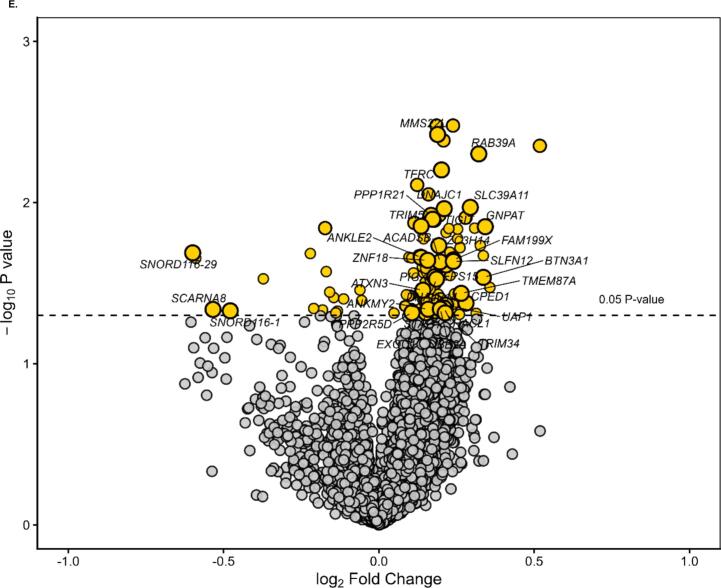
Panel A: Diagram of interleukin-6 (IL-6) levels in plasma before, 3 and 6 h after vaccination against Salmonella Typhii, divided into subjects receiving oxygen therapy and controls. Values are means ± standard error of the mean. Panel B: Volcano plot showing differentially expressed protein markers 6 h after exposure to oxygen therapy with the log p-value on the y-axis and log fold change on the x-axis. Panel C: Diagram of Interleukin-8 (IL-8) protein expression levels before and 6 h after vaccination in subjects receiving oxygen therapy, respectively controls. Values are means ± standard error of the mean. Panel D: Diagram of Brain-Derived Neurotrophic Factor (BDNF) protein expression levels before and 6 h after vaccination in subjects receiving oxygen therapy, respectively controls. Values are means ± standard error of the mean. Panel E: Volcano plot showing differentially genes 6 h after exposure to oxygen therapy with the log p-value on the y-axis and log fold change on the x-axis. Significant changed genes are marked with yellow. Genes included by the factor analysis are marked with gene symbols. (For interpretation of the references to colour in this figure legend, the reader is referred to the web version of this article.)

**Fig. 3:**
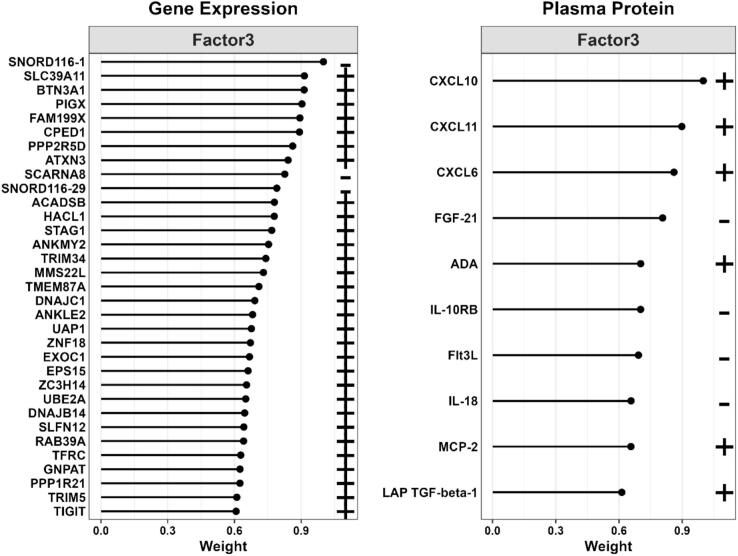
Plot showing relative weight of different genes and proteins in multi-omics factor analysis.

Plasma levels of IL-6 increased after ST-vaccination with a more pronounced rise in subjects receiving oxygen (p < 0.05)(Fig. 2A). A total of 67 immunological protein markers and 9,006 annotated genes were detected in 12, respectively 9 subjects before and 6 h after vaccination (Fig. 1B). As shown in Fig. 2B–D, of 67 immunological protein markers only brain-derived neurotrophic factor (BDNF) was differentially decreased and IL-8 differentially increased by oxygen (p < 0.05 without correction for multiple testing) (Supplementary Table). Gene expression analysis before and 6 h after vaccination showed that 128 genes were differentially expressed by oxygen (p < 0.05 without correction for multiple testing) (Fig. 2E and Supplementary Table). When all immunological proteins were analyzed together with 128 significantly differentially expressed genes without correction for multiple testing, one factor could be identified that significantly captured multi-omic variation related to oxygen exposure after vaccination (Fig. 1B). Prominent features of this factor were increases in the chemokines (CXCL), 6, 10 and 11 (effect size 0.92, standard deviation 0.44, p = 0.048) and a decrease in the expression of small nucleolar RNA (SNORD)116-1 (Fig. 3).

## 4 Discussion

In agreement with our hypothesis the results showed a statistically nominal tendency toward aggravation of systemic inflammation by oxygen therapy in normoxemic healthy subjects. Activation of inflammatory pathways mediated by IL-6 and IL-8 was observed. Additionally, CXCL 6, 10 and 11 were prominent features in the factor analysis explaining the additive effect of oxygen exposure to the experimental inflammatory setting. Furthermore, the multi-omics factor analysis indicated a role for small nucleolar RNA in innate immunity.

Since the pivotal study of oxygen therapy in suspected myocardial infarction [1], guidelines for oxygen therapy in myocardial infarction has changed. Oxygen supplementation is recommended in acute coronary syndromes patients with hypoxaemia (oxygen saturation < 90 %) (Class I, Level of Evidence C). In patients who are not hypoxic (oxygen saturation > 90 %), oxygen therapy is not associated with clinical benefit and is therefore not recommended (Class III, Level of evidence A) [7]. However, in certain clinical scenarios, such as after cardiac arrest, oxygen therapy is still provided liberally to normoxemic patients. Neither did the main results of the DETO2X-study [1] show any harm of oxygen therapy on clinical outcomes, nor did its sub-study of inflammatory markers show any significantly increased systemic inflammation in the oxygen therapy group. However, the sub-study was small and likely influenced by known (medication) and unknown confounders. The current knowledge about inflammation after myocardial infarction indicates that a higher degree of systemic inflammation is associated with a worse prognosis and studies suggest that patients with pronounced systemic inflammation benefit the most from anti-inflammatory treatment [8]. The results of the present study provide additional evidence indicating that even a moderate dose of oxygen given to normoxemic subjects for a relatively short time-period causes enhancement of systemic inflammation and should thus be avoided.

To the best of our knowledge there are no previous studies on the effects of oxygen therapy in moderate doses on the systemic inflammatory response in healthy subjects. However, it is well established that exposure to higher fractions of inspired oxygen causes lung injury characterized by systemic inflammatory response [9]. The underlying mechanism remains undetermined. It can be speculated that it is through interference between oxygen exposure at already low levels and inflammatory cells in the lung stimulating the transcription factor nuclear factor kappa beta that is involved in vaccine-stimulated innate immunity [10]. Interestingly, in our study the small nucleolar RNA SNORD116-1 decreased with oxygen therapy. Accordingly, in the differential expression analysis SNORD116-1 decreased. It is not possible to tell if SNORD-116 is a cause or consequence of the aggravation of inflammation seen in the present study. A role for small nucleolar RNAs in modifying RNA has been implicated in cancer and Prader-Willi syndrome but not in innate immunity [11]. However, one report has implicated a role for the microRNA family 181 in the setting of local inflammation where several microRNAs were down-regulated in inflamed tissue caused by parodontitis [12]. The mechanism behind the association between SNORD-116 and increased inflammatory markers cannot be discerned from the present study. However, it has been shown that small nucleolar RNA decreases transcription of genes [13]. It can thus be speculated that a possible target for SNORD-116 could be the inhibitory protein kappa beta resulting in an increase in active transcription factor nuclear factor kappa beta.

### 4.1 Strengths and limitations

The main strength of the present study is that it was performed in healthy subjects using a well-documented experimental model of inflammation with relevant cardiovascular effects. This minimizes the influence of important confounders that may be present in the acute setting and treatment of myocardial infarction. Furthermore, a novel machine learning method, multi-omics factor analysis, was used. Multi-omics factor analysis has previously been successfully applied to uncover new immunological signatures in acute and chronic coronary syndromes [14].

The main limitation of the study is the limited sample size making correction for multiple testing impossible thereby increasing the risk of a statistical type I error. The results should therefore be considered hypothesis-generating with a need for validation in larger cohorts. Other limitations include that it was limited to men, the treatment of the control group and that a surrogate method for systemic inflammation caused by myocardial infarction was used: First, the study only included men limiting the external validity. The reason for only including men into the study was an additional intervention arm including atorvastatin therapy (data not shown). As statins are contraindicated during pregnancy due to possible teratogenic risks, women of childbearing potential were excluded. Second, the controls received ambient air without an open face mask and thus we cannot exclude that the inflammation was caused by the stress of the face mask and not by oxygen since mental stress has been shown to induce a weak systemic inflammatory response [15]. The relative magnitude of the difference between subjects receiving oxygen and controls was approximately 100 %. It is difficult to find relevant studies to compare with regarding the type of mental stress, but one study of healthy men investigating the effects of the inflammatory response by emergency alarm mobilization in a hospital environment showed that IL-6 increased approximately 60 % suggesting that the results of our study was not entirely caused by the stress of the open face mask [16]. Third, we instead of including patients with myocardial infarction, used an experimental model of inflammation to mimic the inflammation caused by myocardial infarction. However, the model is well-documented with a small rise in IL-6 with a magnitude similar to the rise associated with non-ST-elevation myocardial infarction with cardiovascular consequences, such as endothelial dysfunction, increased vascular stiffness and activation of coagulation factor VII [3,4]. Since vaccination against ST only gives rise to a mild inflammation, the results cannot be extrapolated to larger myocardial infarctions with extensive myocardial injury or reperfusion injury and may thus only be relevant for patients with non-ST elevation myocardial infarction. It can also be questioned if the results from an experimental model of inflammation in healthy men can be extrapolated to myocardial infarction in elderly patients with co-morbidities.

### 4.2 Clinical implications

The results thus reinforce caution with oxygen therapy in normoxemic patients with myocardial infarction. Since it may be harmful, it should be avoided in line with guidelines recommendations [7].

## 5 Conclusions

The results indicate that oxygen therapy enhances experimental systemic inflammation in normoxemic healthy subjects. The mechanism is not clear but future studies should address small nucleolar RNA.

## CRediT authorship contribution statement

**P. Tornvall:** Writing – original draft, Supervision, Project administration, Methodology, Conceptualization. **P. Svensson:** Writing – review & editing, Methodology, Conceptualization. **J. Alfredsson:** Writing – review & editing, Supervision, Methodology, Investigation, Data curation, Conceptualization. **L. Jonasson:** Writing – review & editing, Supervision, Resources, Methodology, Investigation, Conceptualization. **L. Nilsson:** Writing – review & editing, Supervision, Project administration, Methodology, Investigation, Data curation, Conceptualization. **R. Hofmann:** Writing – review & editing, Supervision, Methodology, Funding acquisition, Conceptualization. **SK. Merid:** Software, Writing – review & editing, Data curation, Formal analysis, Conceptualization.

## Funding

The study was supported by the Swedish Heart and Lung Foundation (2021–0273) and the Region Stockholm (RS 2021–0933).

## Declaration of competing interest

The authors declare the following financial interests/personal relationships which may be considered as potential competing interests: Robin Hofmann reports institutional lecture and advisory board fees from MSD/Pfizer and AstraZeneca. Other authors report no conflict of interest.
