# Supplementary material for: Oxygen therapy enhances the systemic inflammatory response in a human model of experimental inflammation
Source: Int J Cardiol Heart Vasc. 2025 Nov 28;62:101846. doi: 10.1016/j.ijcha.2025.101846 (PMC12703976; doi:10.1016/j.ijcha.2025.101846)
Supplement: Supplementary Data 1 [file mmc1.docx]

| Table.1 Differential protein expression analysis of 6-hour expression in oxygen therapy and control groups, adjusted for baseline expression; protein significant at nominal p-value < 0.05. | | | | | | |
| --- | --- | --- | --- | --- | --- | --- |
| Protein | **logFC** | **AveExpr** | **StdError** | **CI_lower** | **CI_upper** | **P.Value** |
| IL-8 | 0,91441 | -0,23799 | 0,353844 | 0,220876 | 1,607945 | 0,017303 |
| BDNF | -0,78715 | 0,137568 | 0,357826 | -1,48849 | -0,08581 | 0,04194 |

| Table.2 Differential gene expression analysis of 6-hour expression in oxygen therapy and control groups, adjusted for baseline expression; genes significant at nominal p-value < 0.05. | | | | | | | | |
| --- | --- | --- | --- | --- | --- | --- | --- | --- |
| Probe.set_ID | **Gene.symbol** | **EntrezGeneID** | **logFC** | **AveExpr** | **StdError** | **CI_lower** | **CI_upper** | **P.Value** |
| TC01002315.hg.1 | MUL1 | 79594 | 0,238656 | 6,243333 | 0,070572 | 0,100335 | 0,376976 | 0,00332 |
| TC18000402.hg.1 | ABHD3 | 171586 | 0,185569 | 7,265238 | 0,054876 | 0,078013 | 0,293125 | 0,00332 |
| TC06001959.hg.1 | MMS22L | 253714 | 0,189008 | 5,465238 | 0,056861 | 0,077561 | 0,300455 | 0,00378 |
| TC06001195.hg.1 | C6orf70 | 55780 | 0,208091 | 6,662381 | 0,063337 | 0,083949 | 0,332232 | 0,00411 |
| TC01002287.hg.1 | PADI2 | 11240 | 0,51817 | 7,181429 | 0,15941 | 0,205725 | 0,830614 | 0,00444 |
| TC11000976.hg.1 | RAB39A | 54734 | 0,321681 | 5,770952 | 0,10058 | 0,124545 | 0,518817 | 0,00498 |
| TC03002155.hg.1 | TFRC | 7037 | 0,201598 | 7,353333 | 0,065163 | 0,073879 | 0,329318 | 0,00626 |
| TC20000708.hg.1 | NAPB | 63908 | 0,123238 | 5,768095 | 0,041138 | 0,042607 | 0,203869 | 0,00776 |
| TC19002685.hg.1 | ZNF564 | 163050 | 0,159938 | 6,055238 | 0,054527 | 0,053065 | 0,266811 | 0,00888 |
| TC17001842.hg.1 | SLC39A11 | 201266 | 0,294326 | 7,831905 | 0,103374 | 0,091713 | 0,496938 | 0,0107 |
| TC10001097.hg.1 | DNAJC1 | 64215 | 0,210387 | 7,760476 | 0,074119 | 0,065114 | 0,355659 | 0,0109 |
| TC02000303.hg.1 | PPP1R21 | 129285 | 0,167926 | 7,198095 | 0,060105 | 0,050121 | 0,285731 | 0,01199 |
| TC09000411.hg.1 | SPIN1 | 10927 | 0,194609 | 8,415714 | 0,069658 | 0,058078 | 0,331139 | 0,012 |
| TC02001089.hg.1 | DNAJC10 | 54431 | 0,278916 | 9,660476 | 0,100398 | 0,082135 | 0,475697 | 0,01241 |
| TC03000588.hg.1 | TIGIT | 201633 | 0,173105 | 5,89619 | 0,06254 | 0,050527 | 0,295683 | 0,01268 |
| TC01003813.hg.1 | ANGEL2 | 90806 | 0,179335 | 7,029524 | 0,064998 | 0,05194 | 0,30673 | 0,01292 |
| TC13000681.hg.1 | DHRS12 | 79758 | 0,114083 | 5,723333 | 0,041582 | 0,032582 | 0,195583 | 0,01335 |
| TC11001341.hg.1 | TRIM5 | 85363 | 0,135879 | 6,680952 | 0,049928 | 0,038019 | 0,233738 | 0,014 |
| TC01001915.hg.1 | GNPAT | 8443 | 0,342513 | 8,998095 | 0,126017 | 0,095518 | 0,589507 | 0,0141 |
| TC06004062.hg.1 | NQO2 | 4835 | -0,17311 | 5,72619 | 0,063912 | -0,29838 | -0,04784 | 0,01439 |
| TC14001141.hg.1 | ERO1L | 30001 | 0,308245 | 13,52238 | 0,113817 | 0,085163 | 0,531327 | 0,0144 |
| TC19001219.hg.1 | STX10 | 8677 | 0,225432 | 7,289524 | 0,083329 | 0,062107 | 0,388758 | 0,01449 |
| TC11000795.hg.1 | SPCS2 | 9789 | 0,253554 | 10,60238 | 0,093865 | 0,069579 | 0,437528 | 0,01461 |
| TC14001063.hg.1 | SEC23A | 10484 | 0,216471 | 8,97381 | 0,080802 | 0,058099 | 0,374843 | 0,01532 |
| TC10000192.hg.1 | RAB18 | 22931 | 0,253465 | 10,09857 | 0,096101 | 0,065106 | 0,441824 | 0,01673 |
| TC08002616.hg.1 | MRPS28 | 28957 | 0,143928 | 6,818571 | 0,054596 | 0,036921 | 0,250936 | 0,01677 |
| TC09000170.hg.1 | NUDT2 | 318 | 0,326744 | 5,83381 | 0,126127 | 0,079534 | 0,573954 | 0,01846 |
| TC14000536.hg.1 | ZC3H14 | 79882 | 0,19321 | 7,925714 | 0,074596 | 0,047002 | 0,339418 | 0,01848 |
| TC20000384.hg.1 | CSE1L | 1434 | 0,261264 | 10,66524 | 0,101428 | 0,062464 | 0,460063 | 0,01904 |
| TC05000301.hg.1 | CDK7 | 1022 | 0,228398 | 7,23619 | 0,08973 | 0,052528 | 0,404268 | 0,02029 |
| TC15000071.hg.1 | SNORD116-29 | 100033821 | -0,59964 | 9,304762 | 0,236002 | -1,0622 | -0,13707 | 0,02049 |
| TC22000846.hg.1 | RRP7A | 27341 | -0,22071 | 5,786667 | 0,087031 | -0,39129 | -0,05013 | 0,0207 |
| TC04001608.hg.1 | ANAPC10 | 10393 | 0,1351 | 5,678571 | 0,053392 | 0,030452 | 0,239747 | 0,02094 |
| TC0X001516.hg.1 | BCAP31 | 10134 | 0,127374 | 7,769048 | 0,050423 | 0,028544 | 0,226203 | 0,02113 |
| TC06001921.hg.1 | SNORD50A | 26799 | 0,336378 | 10,27905 | 0,133415 | 0,074884 | 0,597873 | 0,02134 |
| TC01000074.hg.1 | DFFB | 1677 | 0,096642 | 6,356667 | 0,038497 | 0,021187 | 0,172097 | 0,02183 |
| TC12002155.hg.1 | ANKLE2 | 23141 | 0,136645 | 7,099048 | 0,054474 | 0,029876 | 0,243415 | 0,02192 |
| TC05001660.hg.1 | FBXL17 | 64839 | 0,105859 | 7,742857 | 0,04224 | 0,023069 | 0,188649 | 0,02202 |
| TC02001213.hg.1 | SNORD51 | 26798 | -0,58958 | 10,30381 | 0,235495 | -1,05115 | -0,12801 | 0,02214 |
| TC0X000510.hg.1 | FAM199X | 139231 | 0,239894 | 8,038095 | 0,096258 | 0,051228 | 0,428561 | 0,02267 |
| TC17001144.hg.1 | ZNF18 | 7566 | 0,157015 | 5,512857 | 0,063076 | 0,033387 | 0,280644 | 0,02281 |
| TC17001370.hg.1 | SLFN12 | 55106 | 0,239799 | 6,287619 | 0,096619 | 0,050426 | 0,429173 | 0,02316 |
| TC10000896.hg.1 | ACADSB | 36 | 0,196836 | 7,731429 | 0,079411 | 0,04119 | 0,352482 | 0,02332 |
| TC22000187.hg.1 | CCDC117 | 150275 | 0,238666 | 9,109524 | 0,096653 | 0,049225 | 0,428106 | 0,02378 |
| TC01002649.hg.1 | EPS15 | 2060 | 0,215718 | 12,3581 | 0,087763 | 0,043702 | 0,387734 | 0,02434 |
| TC16000872.hg.1 | ZC3H7A | 29066 | 0,140141 | 8,697619 | 0,057336 | 0,027763 | 0,252519 | 0,02504 |
| TC22000814.hg.1 | SLC25A17 | 10478 | 0,15079 | 5,777619 | 0,061965 | 0,02934 | 0,272241 | 0,0256 |
| TC11000912.hg.1 | MED17 | 9440 | 0,175593 | 7,607619 | 0,072231 | 0,03402 | 0,317165 | 0,02573 |
| TC07000641.hg.1 | TRIM56 | 81844 | -0,16949 | 6,435714 | 0,070284 | -0,30724 | -0,03173 | 0,02679 |
| TC04002945.hg.1 | HSD17B11 | 51170 | 0,144011 | 16,3381 | 0,059912 | 0,026584 | 0,261438 | 0,02722 |
| TC0X000904.hg.1 | CXorf23 | 256643 | 0,111301 | 5,753333 | 0,04633 | 0,020493 | 0,202108 | 0,0273 |
| TC07001452.hg.1 | ERV3-1 | 2086 | 0,17799 | 6,167619 | 0,07443 | 0,032107 | 0,323873 | 0,02792 |
| TC12000098.hg.1 | DSTNP2 | 171220 | 0,229659 | 5,928571 | 0,096318 | 0,040876 | 0,418441 | 0,02832 |
| TC03003368.hg.1 | PIGX | 54965 | 0,179736 | 6,432381 | 0,075672 | 0,031418 | 0,328054 | 0,02886 |
| TC13000454.hg.1 | ZMYM5 | 9205 | 0,187045 | 7,613333 | 0,078806 | 0,032585 | 0,341506 | 0,02896 |
| TC06000186.hg.1 | BTN3A1 | 11119 | 0,336462 | 10,45952 | 0,141894 | 0,05835 | 0,614573 | 0,02909 |
| TC06001121.hg.1 | MIR548U | 100422884 | -0,37213 | 7,411905 | 0,157604 | -0,68103 | -0,06322 | 0,0297 |
| TC04001417.hg.1 | DNAJB14 | 79982 | 0,185439 | 8,438571 | 0,078605 | 0,031373 | 0,339505 | 0,02982 |
| TC08000547.hg.1 | DECR1 | 1666 | 0,166058 | 7,111429 | 0,070806 | 0,027278 | 0,304838 | 0,03068 |
| TC03003399.hg.1 | TRIM59 | 286827 | 0,186245 | 7,248571 | 0,079414 | 0,030594 | 0,341895 | 0,03068 |
| TC02000374.hg.1 | SLC1A4 | 6509 | 0,149575 | 5,92381 | 0,063919 | 0,024293 | 0,274857 | 0,031 |
| TC12000958.hg.1 | RNF34 | 80196 | 0,182766 | 7,847143 | 0,07832 | 0,029258 | 0,336274 | 0,03141 |
| TC13000474.hg.1 | MICU2 | 221154 | 0,184332 | 7,551429 | 0,079293 | 0,028918 | 0,339747 | 0,03199 |
| TC01004026.hg.1 | OPN3 | 23596 | 0,155411 | 6,604286 | 0,066868 | 0,02435 | 0,286473 | 0,03202 |
| TC11000190.hg.1 | ZBED5-AS1 | 729013 | 0,357493 | 6,881905 | 0,1554 | 0,052909 | 0,662077 | 0,0336 |
| TC18000461.hg.1 | TPGS2 | 25941 | 0,164809 | 8,769524 | 0,072041 | 0,023608 | 0,30601 | 0,03447 |
| TC10000052.hg.1 | RBM17 | 84991 | -0,061 | 6,353333 | 0,026728 | -0,11339 | -0,00861 | 0,03485 |
| TC14001446.hg.1 | ATXN3 | 4287 | 0,141851 | 5,598571 | 0,06219 | 0,019959 | 0,263743 | 0,03495 |
| TC17000022.hg.1 | SRR | 63826 | -0,15851 | 5,919524 | 0,06991 | -0,29554 | -0,02149 | 0,03592 |
| TC15001263.hg.1 | TMEM87A | 25963 | 0,264961 | 9,222857 | 0,117299 | 0,035055 | 0,494867 | 0,03654 |
| TC09000492.hg.1 | NCBP1 | 4686 | 0,139796 | 8,700476 | 0,061903 | 0,018465 | 0,261127 | 0,03658 |
| TC08001312.hg.1 | TRAM1 | 23471 | 0,243426 | 14,40238 | 0,107921 | 0,031901 | 0,454952 | 0,03678 |
| TC06002122.hg.1 | SLC18B1 | 116843 | 0,191386 | 7,425714 | 0,084997 | 0,024792 | 0,357981 | 0,03707 |
| TC13000107.hg.1 | EEF1DP3 | 196549 | 0,088698 | 5,765714 | 0,039445 | 0,011385 | 0,166011 | 0,0373 |
| TC01000381.hg.1 | RCC1 | 1104 | 0,10276 | 5,809048 | 0,045783 | 0,013025 | 0,192495 | 0,03761 |
| TC06004058.hg.1 | RNASET2 | 8635 | 0,098902 | 6,83 | 0,044141 | 0,012386 | 0,185419 | 0,0379 |
| TC10001282.hg.1 | TIMM23 | 100287932 | 0,158088 | 11,04905 | 0,070893 | 0,019138 | 0,297037 | 0,03872 |
| TC09000827.hg.1 | MIR4479 | 100616480 | -0,14528 | 6,717619 | 0,065182 | -0,27304 | -0,01752 | 0,03881 |
| TC10001626.hg.1 | NT5C2 | 22978 | 0,196922 | 9,511905 | 0,088437 | 0,023585 | 0,370258 | 0,03898 |
| TC10001002.hg.1 | GDI2 | 2665 | 0,199802 | 16,5619 | 0,089785 | 0,023823 | 0,375781 | 0,03908 |
| TC11000562.hg.1 | TTC9C | 283237 | 0,144229 | 7,66381 | 0,064861 | 0,0171 | 0,271357 | 0,03921 |
| TC04001171.hg.1 | NFXL1 | 152518 | 0,189017 | 6,42381 | 0,085034 | 0,02235 | 0,355683 | 0,03928 |
| TC17001930.hg.1 | CBX4 | 8535 | -0,1135 | 6,391905 | 0,051145 | -0,21374 | -0,01325 | 0,03957 |
| TC07002014.hg.1 | ACTR3C | 653857 | 0,140527 | 5,442381 | 0,06334 | 0,016381 | 0,264672 | 0,03961 |
| TC11000824.hg.1 | ACER3 | 55331 | 0,184796 | 8,164286 | 0,083566 | 0,021007 | 0,348585 | 0,04019 |
| TC19000090.hg.1 | HDGFRP2 | 84717 | -0,05528 | 5,870476 | 0,025037 | -0,10435 | -0,0062 | 0,04047 |
| TC07001617.hg.1 | PON2 | 5445 | 0,143646 | 6,036667 | 0,065219 | 0,015817 | 0,271475 | 0,0409 |
| TC13001720.hg.1 | BIVM | 54841 | 0,152774 | 5,702381 | 0,069389 | 0,016771 | 0,288777 | 0,04097 |
| TC01002796.hg.1 | PIGK | 10026 | 0,269515 | 9,94381 | 0,122851 | 0,028728 | 0,510302 | 0,04162 |
| TC07000735.hg.1 | CPED1 | 79974 | 0,280566 | 5,94619 | 0,128165 | 0,029362 | 0,531769 | 0,04201 |
| TC11002069.hg.1 | RAB6A | 5870 | 0,182007 | 11,37952 | 0,083208 | 0,018919 | 0,345095 | 0,04216 |
| TC13000119.hg.1 | RFC3 | 5983 | 0,239247 | 6,154286 | 0,109743 | 0,02415 | 0,454343 | 0,04277 |
| TC01001422.hg.1 | UAP1 | 6675 | 0,21467 | 6,209048 | 0,0987 | 0,021217 | 0,408123 | 0,04321 |
| TC08000724.hg.1 | TRMT12 | 55039 | 0,211201 | 6,762857 | 0,097192 | 0,020705 | 0,401697 | 0,04337 |
| TC01000934.hg.1 | STXBP3 | 6814 | 0,221941 | 9,598571 | 0,102331 | 0,021371 | 0,422511 | 0,04373 |
| TC03000285.hg.1 | ARIH2 | 10425 | 0,082493 | 7,480952 | 0,038044 | 0,007926 | 0,15706 | 0,04378 |
| TC16000029.hg.1 | FAM173A | 65990 | 0,084936 | 5,941905 | 0,0392 | 0,008103 | 0,161769 | 0,04392 |
| TC19000677.hg.1 | C5AR2 | 27202 | 0,197805 | 5,66619 | 0,091389 | 0,018683 | 0,376927 | 0,04412 |
| TC03003405.hg.1 | CHST2 | 9435 | 0,16077 | 5,627143 | 0,07468 | 0,014398 | 0,307143 | 0,04515 |
| TC03003336.hg.1 | HACL1 | 26061 | 0,210066 | 7,471905 | 0,097585 | 0,0188 | 0,401332 | 0,04516 |
| TC04000152.hg.1 | MED28 | 80306 | 0,233653 | 8,179048 | 0,108553 | 0,020889 | 0,446417 | 0,04518 |
| TC16000250.hg.1 | MIR548D2 | 693131 | -0,20985 | 18,15952 | 0,09759 | -0,40113 | -0,01858 | 0,04536 |
| TC12000222.hg.1 | AEBP2 | 121536 | 0,104578 | 8,791429 | 0,048633 | 0,009257 | 0,199899 | 0,04537 |
| TC02001379.hg.1 | CAB39 | 51719 | 0,133281 | 11,13238 | 0,062004 | 0,011754 | 0,254808 | 0,04544 |
| TC06002000.hg.1 | CD164 | 8763 | 0,220769 | 13,83095 | 0,102737 | 0,019405 | 0,422133 | 0,0455 |
| TC14000605.hg.1 | PAPOLA | 10914 | 0,219071 | 13,97095 | 0,101987 | 0,019176 | 0,418966 | 0,04557 |
| TC0X000587.hg.1 | UBE2A | 7319 | 0,197828 | 7,521905 | 0,092177 | 0,017161 | 0,378494 | 0,04574 |
| TC11001215.hg.1 | ACAD8 | 27034 | 0,108361 | 5,398571 | 0,050502 | 0,009378 | 0,207344 | 0,04579 |
| TC07001161.hg.1 | ANKMY2 | 57037 | 0,158782 | 6,110952 | 0,074042 | 0,01366 | 0,303904 | 0,04589 |
| TC09000934.hg.1 | SCARNA8 | 677776 | -0,53412 | 10,96429 | 0,249211 | -1,02258 | -0,04567 | 0,04601 |
| TC20000816.hg.1 | NDRG3 | 57446 | 0,205757 | 10,27 | 0,09602 | 0,017558 | 0,393956 | 0,04604 |
| TC02001943.hg.1 | FBXO48 | 554251 | -0,18144 | 5,501429 | 0,084813 | -0,34767 | -0,0152 | 0,04637 |
| TC04001725.hg.1 | NEK1 | 4750 | 0,103498 | 6,870952 | 0,048427 | 0,008581 | 0,198415 | 0,04656 |
| TC14001302.hg.1 | ENTPD5 | 957 | 0,111096 | 5,47 | 0,052061 | 0,009057 | 0,213135 | 0,04686 |
| TC15000048.hg.1 | SNORD116-1 | 100033413 | -0,47861 | 11,91286 | 0,22435 | -0,91834 | -0,03889 | 0,04692 |
| TC03001812.hg.1 | STAG1 | 10274 | 0,181048 | 8,389048 | 0,084915 | 0,014614 | 0,347482 | 0,04703 |
| TC01001871.hg.1 | ZNF678 | 339500 | -0,13329 | 5,925238 | 0,062546 | -0,25588 | -0,0107 | 0,04713 |
| TC09000400.hg.1 | CTSL1 | 1514 | 0,201649 | 5,75381 | 0,094799 | 0,015843 | 0,387455 | 0,04749 |
| TC06000591.hg.1 | PPP2R5D | 5528 | 0,106377 | 6,372857 | 0,050182 | 0,008021 | 0,204734 | 0,04818 |
| TC04000324.hg.1 | EXOC1 | 55763 | 0,151543 | 7,421905 | 0,071496 | 0,011411 | 0,291674 | 0,0482 |
| TC15000153.hg.1 | APBA2 | 321 | -0,13861 | 5,674762 | 0,065426 | -0,26684 | -0,01037 | 0,0483 |
| TC11003442.hg.1 | TRIM34 | 53840 | 0,213507 | 7,230952 | 0,100797 | 0,015945 | 0,411068 | 0,04833 |
| TC0X000256.hg.1 | WDR13 | 64743 | 0,048711 | 5,194762 | 0,023016 | 0,0036 | 0,093823 | 0,0485 |
| TC06000144.hg.1 | MRS2 | 57380 | 0,313152 | 7,959048 | 0,148052 | 0,02297 | 0,603334 | 0,04862 |
| TC05003405.hg.1 | SKP1 | 6500 | 0,135632 | 6,591905 | 0,064167 | 0,009864 | 0,261399 | 0,04876 |
| TC03002141.hg.1 | ACAP2 | 23527 | 0,180719 | 11,22286 | 0,085703 | 0,012741 | 0,348696 | 0,04925 |
| TC13000330.hg.1 | GPR180 | 160897 | 0,25934 | 6,629524 | 0,123056 | 0,01815 | 0,500531 | 0,04936 |
| TC12001331.hg.1 | TM7SF3 | 51768 | 0,209974 | 8,747143 | 0,099873 | 0,014223 | 0,405725 | 0,04985 |
